# Surgery

**Published:** 1913-03

**Authors:** E. W. Hey Groves


### SURGERY.

**On the transplantation of tissues and organs.**—The removal of diseased and mutilated organs has for a long time been the chief function of the surgeon's art. But modern methods have opened up a far more ambitious object, viz. the replacement of these lost parts by the transplantation of living tissues taken either from the patient or from another individual. At first this attempt was only applied to skin defects, but step by step its scope has been widened, and it may be worth while, therefore, to review shortly what has been accomplished.

As regards the general principles which underlie this subject of the transplantation of tissues, there are two questions of fundamental importance to be considered. The first relates to the donor of the graft. This may be, and in human surgery generally is, the patient himself. This is known as an auto-plastic graft, and there can be no doubt that this method gives the best chances of success. In such an auto-plastic transplantation there are only two factors necessary for success, these being an aseptic healing of the graft and a satisfactory blood supply which will ensure its final preservation. The ordinary Thiersch's skin grafting is the commonest example of this, and its applicability is only limited by the conditions named. The largest areas divested of skin can be covered, but as the graft only consists of superficial skin layers, it will not reproduce hair or sweat glands. Unfortunately in one of the commonest conditions where it is desirable to make new skin grow, neither of the conditions can be fulfilled. I refer to the large chronic ulcers of the leg. The bed for the graft can in this region never be rendered aseptic, and more important than this, the circulation is so poor that new tissue growth is almost impossible. Hence any but the smallest grafts in this region constantly fail. Then the graft may be obtained from another individual, *i.e.* from an animal of the same species. This is known as a homo-plastic graft. Under these conditions another important factor comes into play. The cells and tissues of one individual do not necessarily live in the blood stream of another. In fact, it is held by many pathologists that they never do so. Clinically, of course, there can be no doubt that the homo-plastic graft does often fulfil its function, but this may be that it merely forms a scaffolding upon which the tissues of the recipient of the graft builds up a new structure.

The simplest form of homo-plastic grafting is that of the immediate transfusion of blood. Now whatever be the actual fate of the transferred corpuscles and plasma, there can be no doubt that the new blood can functionate for a sufficient time to allow the restoration of all vital functions. Some of the most striking instances of this have occurred after severe hemorrhage in new-born infants when the blood has been supplied by the father of the child. A remarkable and dramatic account of such a case was given by Lambert, Carrel and Brewer.[1]

Schöne, who has summarised much of the recent work on transplantation[2] and written a short abstract of the subject in the December *Jahreskurse für Arztliche Fortbildung*, 1912, lays great stress upon the factor of blood relationship in the success of homo-plastic grafting. And all the most remarkable successes in direct blood transfusion took place when the blood was given from parent to child or *vice versa*.

The third type of graft is the hetero-plastic, in which the graft is taken not only from another animal but from one of a different species. In such a case there can be no doubt whatever that the graft quickly dies, but nevertheless it is substituted by new tissue, and therefore may actually lead to a perfect restoration of function. In this type of transplantation the grafting is often little more than the use of an organic easily absorbable splint.

The second fundamental consideration is the question as to how far the graft really lives and how far it is merely replaced by the tissue cells of its host. Strictly speaking, it is difficult accurately to define what we mean by "living." Take the question of the transfused blood cells. We know that blood cells under healthy conditions are constantly being destroyed and replaced, so that if it could be proved that transfused blood cells quickly die, it would not be fair, therefore, to say that they had not lived sufficiently long to carry on the vital function in their new host. Thus the question of vitality is rather a relative one. All tissue cells are constantly undergoing destruction and renewal, and it is no matter if the tissues of the graft have a comparatively short life, provided that they can ensure the renewal of similar tissue from the host. This substitution of the tissues of the graft by those of the host has been fully worked out by experimental observation by Axhausen,[3] Rehn,[4] and Wrede.[5]

It must suffice for the purposes of this summary to indicate some of the most useful applications of these new methods and then give a few examples of the more startling of its developments.

*Some practical applications of grafting.*—König has reproduced the ala nasi by transplanting a piece of the ear with its cartilage.

1 *Med. Rec.*, 1908, lxxiii. 885.

2 *Die Homoplastische and heteroplastische Transplantationen.* Springer, Leipzig, 1912.

3 *Arch. f. klin. Chir.*, 1909, lxxxviii. 23; 1912, xcix. 519.

4 *Beitr. z. klin. Chir.*, Bd. lxix.; *Arch. f. klin. Chir.*, 1912, xcviii. 1.

5 *Verhandl. d. deutsch. Gesellsch. Chir.*, 1909, p. 35.

Plange among others has replaced an opaque cornea by grafts from recently removed eyes.[1] The transplantation of fascia has had many and diverse uses[2]; of these the most important are the filling-in of defects in the abdominal wall associated with large hernias and the replacement of large areas of dura mater removed in the treatment of tumours of the brain. The fascia is taken from the fascia lata of the thigh. Bones are frequently transplanted for the purpose of filling up the gap left after the removal of tuberculous or myelomatous disease.[3] This is done either by taking large pieces of bone, such as the shaft of the fibula, *en masse* or by simply filling up a gap by bone chips.

*Some remarkable instances of grafting and isolation of viscera.*—A few instances stand out as illustrating the extreme possibilities of these methods, though for practical reasons their applicability can never be great. Kuttner[4] replaced the head and upper part of the shaft of the femur by the parts taken from a recently defunct corpse. Union took place and a functional joint resulted. Perhaps the most perfectly attested case of joint transplantation was that shown by Lexer three years after the operation at the German Surgical Congress. A girl of 18 had a tuberculous knee excised—replaced by the corresponding parts from a limb which had been freshly amputated.[5] A strong and flexible joint resulted, and the patient was shown walking three years later, the new parts having been perfectly incorporated in the old.

The latest experiment of that master pioneer in this field, Dr. Alexis Carrel, has been related by Pozzi.[6] In an etherised cat, after preliminary ligature of all the great vessels entering and leaving the body cavities, the whole of the thoracic and abdominal viscera were removed in one mass and placed in Ringer's solution at a temperature of 38° C. Without any artificial assistance, the heart continued to beat for thirteen hours, and the activity of the alimentary canal continued without interruption. In fact, the whole mass of viscera continued to live and functionate as an independent organism, without the control of any central nervous system.

E. W. Hey Groves.

[1] *Klin. Monatsbl. f. Augenh.*, Bd. xlvi.

[2] See Kirschner, *Beitr. z. klin. Chir.*, 1909, lxv. 472, and *Arch. f. klin. Chir.*, 1910, xcii. 888.

[3] Macewen, *The Growth of Bone*, 1912; Axhausen, *loc. cit.*

[4] *Beitr. z. klin. Chir.*, Bd. lxxv.

[5] *Arch. f. klin. Chir.*, Bd. lxxx. lxxxvii.; *Verhandl. d. deutsch Gesellsch. f. Chir.*, 1911.

[6] *Bull. Med.*, 1913, xxvii. 27–28,.

